# Sickle Cell Anemia and Inflammation: A Review of Stones and Landmarks Paving the Road in the Last 25 Years

**DOI:** 10.3390/hematolrep17010002

**Published:** 2025-01-10

**Authors:** Jessica Dorneles, Amanda de Menezes Mayer, José Artur Bogo Chies

**Affiliations:** Laboratory of Immunobiology and Immunogenetics, Post Graduation Program in Genetics and Molecular Biology (PPGBM), Universidade Federal do Rio Grande do Sul (UFRGS), Porto Alegre 91501-970, Brazil; jessicadornelesrs@gmail.com (J.D.); amenezes.mayer@gmail.com (A.d.M.M.)

**Keywords:** sickle cell anemia, inflammation, vaso-occlusion, mannose-binding lectin, adhesion molecules

## Abstract

A quarter of a century ago, sickle cell disease (SCD) was mainly viewed as a typical genetic disease inherited as a classical Mendelian trait. Therefore, the main focus concerning SCD was on diagnosis, meaning, genotyping, and identification of homozygous and heterozygous individuals carrying the relevant HbS mutant allele. Nowadays, it is well established that sickle cell disease is indeed the result of homozygosis for the HbS variant, although this single feature is not capable of explaining the highly diverse clinical presentation of SCD. In fact, an important feature of SCD is the chronic inflammation that accompanies the sickling of erythrocytes. In this manuscript, we will revisit the early evidence of inflammation in SCD and review what was uncovered during the last 25 years. Here, we describe Sickle cell anemia as a major participant in the history of science. In fact, SCD was the first genetic disease where the causal mutation was identified and is also the first disease for which treatment through genome editing was approved, making this disease a landmark in the road of molecular biology.

## 1. Introduction

**Figure 1:**
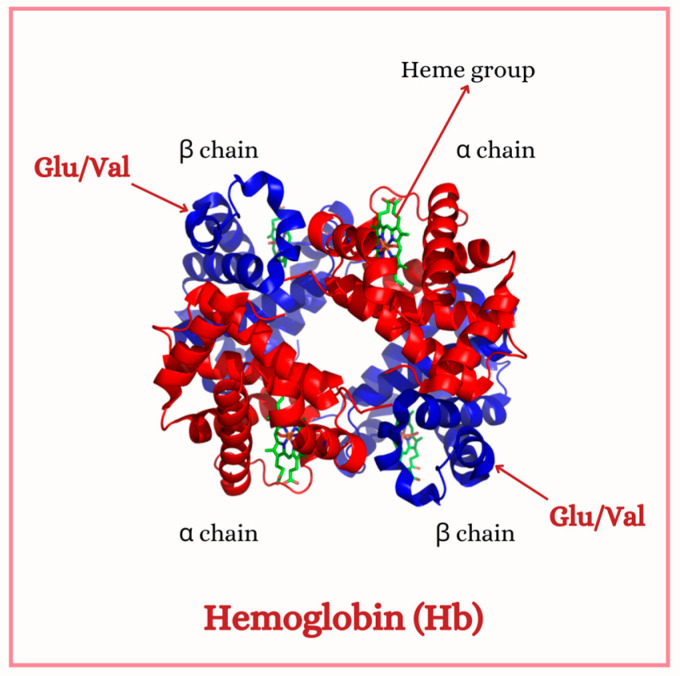
Hemoglobin (Hb) molecule. Normal hemoglobin have a glutamic acid coded at the codon 6, while hemoglobin S have a valine at the same point. This figure was generated using Canva (Canva for Windows is available in https://www.canva.com). The contents used are copyright-free.

Sickle cell anemia is a hereditary genetic disorder caused by a mutation in the beta-globin gene, resulting in the synthesis of hemoglobin S (HbS), an abnormal variant of hemoglobin (Figure 1). This mutation involves a substitution of adenine for thymine, leading to the replacement of glutamic acid by valine at position six of the beta-globin chain. This change alters both the structure and solubility of the protein, promoting its polymerization at low oxygen tension [1].

**Figure 2:**
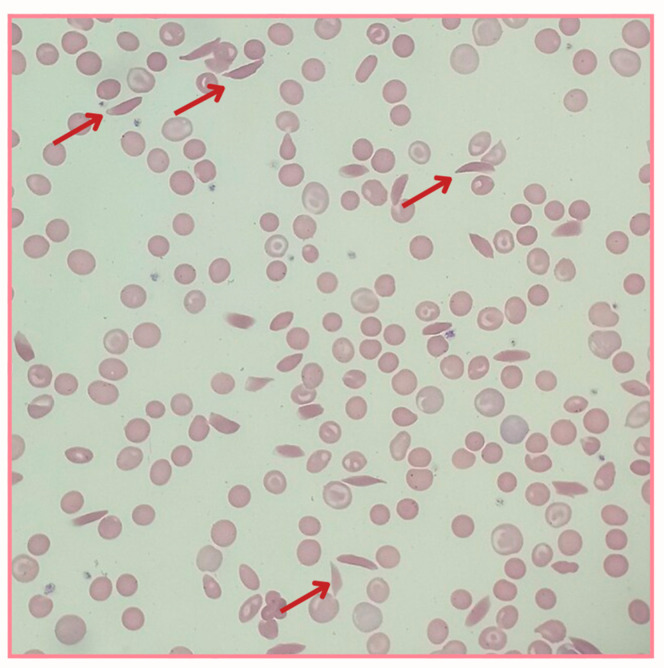
Blood smear from a patient with SCD. Red arrows identify sickle cells, which are crescent-shaped, surrounded by normal erythrocytes. This figure was generated using Canva (Canva for Windows is available in https://www.canva.com). The contents used are copyright-free.

Citologically, erythrocytes, which are responsible for oxygen transport, present normally as a round biconcave cell with a thin and flexible membrane that carries hemoglobin [2]. Nevertheless, when carrying HbS, such cells become rigid and assume a sickle shape as they pass through regions with low oxygen tension, impairing their ability to navigate through blood vessels (Figure 2). As a consequence, these sickled erythrocytes obstruct small vessels, leading to vaso-occlusion, tissue ischemia, and intense pain episodes known as vaso-occlusive crises. Furthermore, chronic hemolysis, caused by the premature destruction of these abnormal erythrocytes, releases free hemoglobin into the plasma, contributing to endothelial dysfunction and a systemic inflammatory response.

Although a deleterious condition in situations of hypoxia, sickle cell disease provides an advantage concerning infection by *Plasmodium* spp. In malaria-endemic regions, sickle-shaped erythrocytes *Plasmodium* spp. infection is difficult since the microenvironment provided by these cells is not favorable to the parasite development [3]. In fact, the most common mutations related to HbS originated and were positively selected in regions where malaria is endemic. Presently, SCD has wide distribution globally, although the sub-Saharan African region still presents one of the most prevalent morbi-mortality rates associated with SCD compared to other territories [4]. Studies estimate that in 2050, there will be more than 400,000 newborns with SCD, with predominance in countries like Nigeria and the Democratic Republic of Congo [5].

**Figure 3:**
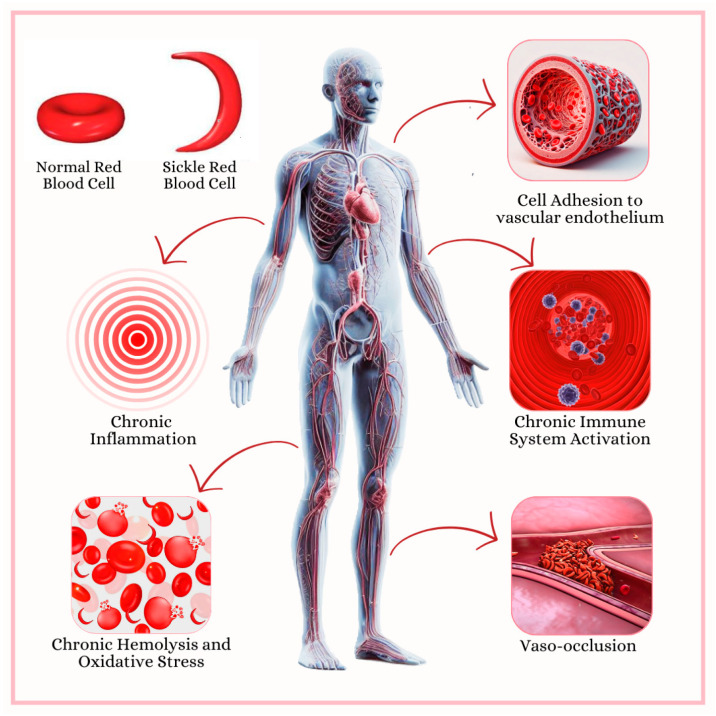
Consequences of sickle cell disease include chronic inflammation, chronic intravascular hemolysis and oxidative stress, cell adhesion to vascular endothelium, constant immune system activation, and vaso-occlusion. Molecules and pathways involved in these conditions are detailed in Table 1. This figure was generated using Copilot and Canva (Canva for Windows is available in https://www.canva.com. Copilot for Windows is available in https://copilot.microsoft.com/). The contents used are copyright-free.

Despite its clinical and epidemiological importance, SCD is a disease that is often poorly studied, probably due to its lower prevalence in developed countries. We consider it essential to understand the several pathways related to chronic inflammation in SCD patients in order to have a better understanding of the features associated with this condition, allowing for the development of targeted clinical and pharmacological procedures. Figure 3 presents a general overview of the mechanisms associated with the chronic inflammatory response present in SCD patients, which will be further addressed.

The first genetic disease was identified at the molecular level in 1949 by Linus Pauling and his colleagues [6], research on SCD up to the 2000s primarily focused on identifying the molecular causes of the symptoms and crises experienced by patients, as well as on the search for effective treatments, which included nitric oxide supplementation and the induction of fetal hemoglobin production through the chemotherapeutic agent hydroxyurea. Over the past 25 years, research in sickle cell anemia has significantly shifted from a mere understanding of vaso-occlusive crises to a deeper investigation into the chronic inflammation associated with the disease. This growing interest reflects the understanding that chronic inflammation plays a central role in the pathogenesis of sickle cell anemia, contributing to its systemic complications [7]. Recent studies have focused on the mechanisms and dynamics of this inflammation, identifying the molecular and cellular pathways involved, and exploring underlying causes such as chronic intravascular hemolysis, free hemoglobin release, endothelial activation, and oxidative stress [8].

These investigations have revealed that inflammation in sickle cell anemia is a continuous process, exacerbated by acute vaso-occlusive episodes and triggered by multiple factors, thereby increasing the complexity and severity of the disease. Here, we will review this concept of inflammation in sickle cell anemia, highlighting recent contributions to the understanding of this disease.

## 2. Sickle Cell Disease—Genetics

The first significant milestone in the study of sickle cell anemia occurred in 1949 when Linus Pauling and his team employed electrophoresis to analyze the physical properties of hemoglobin. They identified a difference in electrophoretic mobility between hemoglobin from healthy individuals (HbA) and that from patients with sickle cell anemia (HbS), indicating a structural alteration in the molecule [6]. This pioneering discovery demonstrated that sickle cell anemia results from a molecular alteration in hemoglobin, laying the groundwork for understanding the disease as a genetic disorder. In the subsequent years, in 1956, Vernon Ingram advanced this understanding by identifying the point mutation in the hemoglobin subunit beta (*HBB*) gene, which codes for the beta chain of hemoglobin, a protein crucial for oxygen transport in the blood [9].

Hemoglobin is composed of four subunits: two alpha chains and two beta chains, with each beta chain encoded by the *HBB* gene. As previously mentioned, the mutation associated with the sickle cell trait is adenine (A) to thymine (T) transversion at nucleotide 20 of the *HBB* gene, resulting in the substitution of the codon GAG, which encodes glutamic acid, to GTG, which encodes valine at position 6 of the beta-globin chain [10]. This substitution alters the chemical composition of the amino acid residue, replacing glutamic acid, a polar and negatively charged amino acid, with valine, a hydrophobic and neutral amino acid. The resulting change in chemical composition induces a modification in the three-dimensional structure of hemoglobin under low oxygen tension, leading to the formation of sickle-shaped cells and the associated disease symptoms. Such polymerization affects erythrocyte deformability, meaning the ability red cells have to adapt their shape, minimizing their resistance to flow. Such stiffer cells have difficulty passing through small blood vessels, leading to blood flow obstruction (vase occlusion), intense pain, and tissue damage. As a consequence, small arteries and capillaries become clogged causing even more local oxygen deficiency.

The Mendelian inheritance of sickle cell anemia was elucidated by James V. Neel and Ernest Beutler in the 1950s, demonstrating that the disease follows an autosomal recessive pattern of inheritance. The disease is clinically expressed only in individuals who are homozygous for the mutant allele (HbSS). Heterozygous carriers (HbAS), who possess one wild type and one mutant allele, do not exhibit symptoms of the disease but can pass the mutant allele to their offspring. This means that both parents must carry the mutant allele for the condition to manifest in their progeny [11].

## 3. Sickle Cell Disease—Inflammation

The association between inflammation and sickle cell anemia started to emerge more clearly from the 2000s onwards. Although inflammation had long been recognized as a component of the disease, it was during this period that more detailed research began to demonstrate the importance of chronic inflammation in the pathogenesis of sickle cell anemia. It was only in 2001 that sickle cell anemia was first directly referred to as a chronic inflammatory disease [7]. Subsequent studies have shown that inflammation plays a crucial role in vaso-occlusive episodes and disease-related complications, with the continuous release of pro-inflammatory cytokines and the activation of inflammatory cells contributing to the pathology of the disease [12]. Below, we will approach specifically some of the important inflammatory molecules and pathways that can interfere with the clinical features of a sickle cell anemia patient.

## 4. Chronic Intravascular Hemolysis Driving Inflammation in SCD

Over the past two decades of scientific advancements, studies have demonstrated that in sickle cell disease, chronic inflammation is triggered by various factors related to the pathology of the disease. Chronic intravascular hemolysis is one of the key factors, as the continuous destruction of sickled erythrocytes releases free hemoglobin and other cellular components into the plasma. This release initiates an inflammatory response, with free hemoglobin acting as a potent inflammatory agent that contributes to endothelial dysfunction and the activation of inflammatory cells [10]. Chronic intravascular hemolysis promotes the activation of endothelial cells lining the blood vessels. This activation leads to the expression of adhesion molecules and the release of pro-inflammatory cytokines, further exacerbating the inflammatory state [8,13].

## 5. Inflammatory Cells and Adhesion Molecules in SCD

In the context of sickle cell disease (SCD), inflammatory cells not only respond to normal stress situations, that is, activated only when necessary, but remain persistently active. This continuous activation is far from their physiological role and contributes to a state of chronic inflammation. Activated leukocytes, including neutrophils, monocytes, and lymphocytes, in response to genetic or environmental stimuli, release inflammatory substances such as cytokines and chemokines. These substances coordinate the immune response; however, when produced in excess, they can lead to exacerbated inflammation [12].

Adhesion molecules are also crucial elements in the pathophysiology of sickle cell disease, facilitating the adhesion of sickled cells and leukocytes to the vascular endothelium. Molecules such as P-selectin, ICAM-1 (intercellular adhesion molecule), and VCAM-1 (vascular adhesion molecule) are often overexpressed in patients with SCD. This overexpression promotes interactions between inflammatory cells and the endothelium, resulting in blood flow obstruction and contributing to vaso-occlusive crises. Thus, the imbalance between pro-inflammatory and anti-inflammatory molecules is a key factor in the severity and recurrence of clinical complications in sickle cell disease [12].

Polymorphisms in genes related to immune response, such as those encoding cytokines, can influence the quantity/levels of these molecules. Tozatto-Maio and collaborators analyzed how single nucleotide polymorphisms (SNPs) in genes encoding inflammatory proteins influence clinical complications in patients with sickle cell disease (SCD) [14]. They studied 20 SNPs in genes related to inflammatory receptors, such as Toll-like receptors (TLR), NK cell receptors (NKG), human leukocyte antigens (HLA), MICA (MHC class I chain-related sequence), and CTLA-4 (cytotoxic T-lymphocyte-associated protein 4).

Among the findings, the SNPs TLR2 rs4696480 and rs3804099, as well as HLA-G rs9380142, were associated with a lower frequency of clinical complications [10]. Additionally, certain SNPs in the NKG2D receptor loci were linked to a reduced incidence of retinopathy [14]. These findings suggest that the cited polymorphisms may offer protection against disease complications, contributing to a better understanding of the inflammatory pathways involved in SCD and paving the way for the identification of new biomarkers that could predict disease severity.

Furthermore, the interactions between different genetic variants may account for the diversity in manifestations of sickle cell disease among patients. Some individuals may possess a genetic predisposition that renders them more susceptible to oxidative stress—a type of cellular damage that occurs when there is an imbalance between free radicals and antioxidants in the body—and vascular damage. This susceptibility may result in more severe complications of the disease [15,16].

On the other hand, control of the chronic inflammation observed in SCD is a potential way to treat the condition. As an example of a molecule reported to play a significant immunomodulatory role, we can cite the transferrin receptor protein 1 (CD71). CD71 is expressed in immature erythrocytes, including reticulocytes, which are notably increased in anemic conditions [17,18]. These reticulocytes are capable of secreting regulatory molecules that suppress T-cell function, thereby contributing to immune tolerance [19,20].

## 6. Cytokine Expression in SCD

Several cytokines are inflammatory modulators that act by favoring or inhibiting the immune response and thus maintaining homeostatic control [21]. Levels of pro-inflammatory cytokines, such as tumor necrosis factor (TNF), interleukin-1 (IL-1), interleukin-8 (IL-8), and prostaglandin E_2_ (PGE_2_), are commonly increased in sickle cell disease [22,23]. These cytokines are related to higher adhesion of sickle cells to the vascular endothelium, contributing to episodes of vaso-occlusion, in addition to aggravating the inflammatory complications of the disease [8].

Patients with SCD have a constant state of platelet activation [24] and their greater adhesion to endothelial tissue was also demonstrated by Proença-Ferreira et al. [25]. Evidence shows that there is an increased production of inflammatory cytokines such as IL-1β, CD258 (TNFSF14), and CD40L (TNFSF5) in SCD platelets. These characteristics of platelet cells are directly associated with inflammation and vaso-occlusion [25,26,27].

Increased leukocyte counts are frequently observed in SCD. This expansion, together with increased adhesion of these cells to the vascular endothelium, is also strongly associated with episodes of vaso-occlusion [28,29]. The granulocyte–macrophage colony-stimulating factor (GM-CSF) is a cytokine that improves the white blood cells’ development, which is involved in the positive regulation of leukocyte count in SCD [30].

It has been demonstrated by Miguel and collaborators that the pro-inflammatory cytokines IL-8 and TNF-α enhance the expression and function of neutrophil adhesion molecules in SCD patients [31]. In contrast, transforming growth factor β1 (TGF-β1) expression can reduce acute microvascular inflammation in addition to suppressing neutrophil adhesion on vascular endothelial tissue, demonstrating that the role of cytokines depends on the context in which they are inserted [32].

The number of eosinophils can be up to three times higher in patients with SCD compared to those without this condition. These cells, when isolated from patients with SCD, demonstrate greater adhesion, in addition to being in a constant state of activation [33]. The study by Pallis et al. (2014) explored the use of hydroxyurea in reducing eosinophil adhesion and degranulation in patients with sickle cell anemia. The main findings demonstrate that hydroxyurea decreases inflammation and eosinophil activation, contributing to the reduction in the inflammatory response and supporting the use of this medication for the reduction in inflammatory complications and vaso-occlusive crises [34].

## 7. Oxidative Stress in SCD

Oxidative stress, generated by the interaction between free hemoglobin, leukocytes, and other cellular components, intensifies inflammation, causing tissue damage and activating additional inflammatory pathways, thereby aggravating the inflammatory condition [35]. Additionally, during vaso-occlusive crises, sickled erythrocytes obstruct small blood vessels, resulting in ischemia and subsequent tissue reperfusion. These events create an intense inflammatory environment, characterized by the release of inflammatory mediators and the recruitment of inflammatory cells. Chronic inflammation is sustained by the continuous release of pro-inflammatory cytokines such as TNF-α, IL-1, and IL-6, which perpetuate the inflammatory state and contribute to the complications associated with the disease [36,37].

Thus, inflammation in sickle cell disease arises from a continuous cycle of intravascular hemolysis, oxidative stress, endothelial activation, and vaso-occlusive crises. This cycle creates an inflammatory environment that exacerbates the disease and its complications, maintaining the inflammatory state and worsening the clinical condition of the patients.

Advances in molecular biology and the growing number of studies on the mechanisms involved in the inflammatory processes of sickle cell anemia have been fundamental to the development of more targeted and effective therapies. With these advances, treatments have emerged that go beyond palliative interventions, acting on the underlying causes of inflammation and reducing associated complications. This approach enables strategies that promote the reduction in crises and significantly improve the quality of life and prognosis of patients, pointing to a future of greater control over the disease.

## 8. Mannose-Binding Lectin in SCD

Mannose-binding lectin (MBL) is a protein formed in the liver that acts on innate immunity, activating the complement system through the opsonization of molecules [38]. Evidence suggests that mannose-binding lectin is involved in the recognition and removal of sickle cells, thus controlling chronic inflammation [39]. In addition, MBL is also associated with infection defense and the control of immune complex deposition, which is especially important during infancy, where the adaptive immune system is still in the process of maturation [40,41].

The MBL2 gene is located in the 10q11.1–q21 chromosome and generates a protein with 248 amino acids [42,43]. Three dominant variants (R52C, G54D, G57E) associated with low serum expression of MBL have been identified in exon 1 of the MBL2 gene, in addition to two variants at the −550 and −221 positions of the promoter region [44]. Heterozygotes for these variants have 5- to 10-fold fewer functional MBL levels [45].

Studies show that MBL2 variant alleles are common in populations, even in healthy individuals. The highest frequencies are found in Africa, where around half of the individuals evaluated have at least one of the three cited alleles with mutation. Among these, the p.G57E allele is predominant in the heterozygous state [38,46].

On the other hand, Zachariah and collaborators (2016) observed that SCD patients who present mutant alleles for MLB2 had a greater rate of microbiological infections, despite the allelic frequencies of MBL2 having no substantial differences between control and SCD groups [47]. Neonato et al. (1999) analyzed 215 SCD children living in Paris, with ancestry from Western Africa, North Equatorial Central Africa, South Equatorial Central Africa, and the French West Indies, and more than 50% of the patients presented mutant MBL genotypes. In addition, approximately 40% of these carriers individuals had previous serious infections, reinforcing the protective role of MBL [39].

Whereas MBL is primarily involved in phagocytosis control through opsonization of molecules, its deficiency could lead to sickle red blood cell accumulation on vessels, evolving to their adhesion on the endothelium and subsequent vaso-occlusive episodes [48,49].

It is clear that MBL plays an essential role in the inflammatory control of SCD, but the scarcity of studies in the area ends up making it difficult to understand the exact mechanisms involved in this pathway and, consequently, restricting the development of new treatments for this condition.

## 9. Final Considerations

The data presented in our study reinforce the central role of inflammation in the pathogenesis of sickle cell disease (diagrammed in Figure 1). Several studies have highlighted that persistent activation of inflammatory cells and the continuous release of pro-inflammatory cytokines play a crucial role in vaso-occlusive episodes and disease-related complications.

Chronic intravascular hemolysis emerges as a key factor in this process, as the continuous destruction of sickle erythrocytes releases free hemoglobin and other cellular components that not only act as potent inflammatory agents into the plasma but also promote endothelial dysfunction and aggravate the inflammatory state by activating inflammatory cells. In addition, oxidative stress, amplified mainly by the interaction between free hemoglobin and leukocytes, intensifies tissue damage and activates additional inflammatory pathways, further aggravating the inflammatory condition. This persistent interaction between inflammatory cells and the endothelium directly contributes to blood flow obstruction and the development of vaso-occlusive crises.

Although research has significantly advanced the understanding of the inflammatory mechanisms associated with SCD in recent years, much remains to be explored. In fact, follow-up of inflammatory cytokines and all other pro-inflammatory molecules and pathways should be included in the clinical practice when we are dealing with an SCD patient, even when typical inflammatory clinical symptoms are absent. Future studies should seek therapeutic strategies that control inflammatory activation and minimize the effects of oxidative stress, with the aim of improving the quality of life of the patients and reducing disease complications.

## Figures and Tables

**Table 1:** Genes and molecules with a potential role on chronic inflammation in SCD.

Molecule/Gene	Function
P-selectin	Adhesion Molecule
Intercellular Adhesion Molecule (ICAM-1)	Adhesion Molecule
Vascular Adhesion Molecule (VCAM-1)	Adhesion Molecule
Tumor Necrosis Factor (TNF)	Cytokine
CD40L (TNFSF5)	Cytokine
CD258 (TNFSF14)	Cytokine
Tumor Necrosis Factor α (TNF-α)	Cytokine
Interleukin-1 (IL-1)	Cytokine
Interleukin-6 (IL-6)	Cytokine
Interleukin-8 (IL-8)	Cytokine
Prostaglandin E_2_ (PGE_2_)	Cytokine
Interleukin-1β (IL-1β)	Cytokine
Granulocyte–Macrophage Colony-Stimulating Factor (GM-CSF)	Cytokine
Mannose-Binding Protein (MBL)-Variants associated to low expression	Innate Immune Response

## Data Availability

No new data were created or analyzed in this study. Data sharing is not applicable to this article.
